# The influence of breeding history, origin and growth type on population structure of barley as revealed by SSR markers

**DOI:** 10.1038/s41598-020-75339-4

**Published:** 2020-11-05

**Authors:** Seyyed Abolghasem Mohammadi, Nayyer Abdollahi Sisi, Behzad Sadeghzadeh

**Affiliations:** 1 Department of Plant Breeding and Biotechnology, Faculty of Agriculture, University of Tabriz, 51666 Tabriz, Iran; 2 Center of Excellence in Cereal Molecular Breeding, University of Tabriz, 51666 Tabriz, Iran; 3 Center for Cell Pathology, Department of Life Sciences, Khazar University, Baku, AZ1096 Azerbaijan; 4 Dryland Research Institute of Iran, Maragheh, Iran

**Keywords:** Biotechnology, Genetics

## Abstract

Natural and mass selection during domestication and cultivation favored particular traits of interest in barley. In the present study, population structure, and genetic relationships among 144 accessions of barley landraces and breeding materials from various countries were studied using a set of 77 and 72 EST-SSR and gSSR markers, respectively distributed on seven chromosomes of barley. In total, 262 and 429 alleles were amplified in 77 EST-SSRs and 72 gSSR loci, respectively. Out of which, 185 private/group-specific alleles were identified in the landraces compared with 14 in "cultivar and advanced breeding lines", indicating the possibility to introgress favorite alleles from landraces into breeding materials. Comparative analysis of genetic variation among breeding materials, Iranian landraces, and exotic landraces revealed higher genetic diversity in Iranian landraces compared with others. A total of 37, 15, and 14 private/group-specific alleles were identified in Iranian landraces, exotic landraces, and breeding materials, respectively. The most likely groups for 144 barley genotypes were three as inferred using model- and distance-based clustering as well as principal coordinate analysis which assigned the landraces and breeding materials into separate groups. The distribution of alleles was found to be correlated with population structure, domestication history and eco-geographical factors. The high allelic richness in the studied set of barley genotype provides insights into the available diversity and allows the construction of core groups based on maximizing allelic diversity for use in barley breeding programs.

## Introduction

Barley is one of the oldest known domesticated crops with a large genome and a high number of varieties and accessions worldwide. Archaeological and molecular studies indicate that it was domesticated from two-row wild barley *Hordeum vulgare* L. subsp. *spontaneum* in the Near East about 10,000 years ago^1–3^. Due to the wide adaptation of barley to various environmental conditions, its germplasm pool with high genetic diversity has the potential to breed for adaptation to different environmental conditions. However, genetic diversity in modern barley cultivars has been declined following intense selection in modern plant breeding programs. Therefore, characterization and the use of barley germplasm arrays such as landraces are essential for identifying candidate genes for important traits^4–6^.

Domestication and human selection in subsistence agriculture over a long period resulted in the development and evolution of landraces with high adaptation to the local natural environments. In such systems, natural selection, seed exchange, and human migration generated a huge genetic variation which has been maintained by farmers^7,8^. Due to heterogeneous nature of the landraces, huge genetic diversity is structured at the field and farmer levels (between and within populations). Morphological and agronomic traits, as well as molecular tools, have been utilized to assess components of the genetic diversity in landraces^9,10^.

Modern plant breeding activities in the recent century resulted in the development of a large number of elite varieties with a higher yield, better quality, and resistance to stresses, but the narrow genetic background^11^. Replacement of highly adapted landraces by modern varieties performing under optimal conditions but failing under harsh environments^12^ resulted in the disappearance of most landraces of crop plants from practical farming^11,13^. Although in many countries, landraces were completely replaced by modern varieties starting from a long time back, in some marginal areas, to take advantage of the specific adaptations of landraces to the agro-ecosystem, their cultivation still continues^14^. The landraces of self-pollinated species such as barley harboring the chromosomal regions consisted of blocks of genes with a low frequency of recombination which may confer a specific adaptation to specific environments^15^. This shows that the landraces are ideal association panels to identify genes controlling adaptive traits in crop species^10,16^. Therefore, for efficient utilization of barley landraces in breeding programs and to develop the strategies for their optimal conservation, it is necessary systematically to evaluate molecular diversity encompassed in the landraces collections^17^. Among the DNA markers, SSR markers have been extensively use to analyze population structure and genetic diversity in barley germplasm, due to the high polymorphism, reproducibility, co-dominant and multi-allelic nature when compared to most of the marker systems^16–22^.

The present study was carried out to (i) characterize the allelic diversity, and population structure in a collection of 119 barley landraces from Iran and across the world including two-row and six-row types with different growth habits and compared with those of barley cultivars and advanced breeding lines and (ii) compare the efficiency of genomic and EST- SSR markers in assessing genetic relationships and population structure.

## Results

### Allelic variation of EST-SSRs and genomic SSRs

Genetic relationships in a set of 144 barley genotypes were investigated using 72 gSSR and 77 EST-SSR loci distributed at seven barley chromosomes. The interval spanning between markers used varied from 5.80 cM in 4H to 8.10 cM in 6H with an average of 6.93 cM. The number of alleles/locus varied from 3.69 (5H) to 6.04 (4H) and PIC ranged from 0.44 (2H) to 0.62 (4H) (Table 1). The used SSR markers were selected based on technical reliability, polymorphism, and their distribution in the barley genome. Description of the EST-SRR and gSSR loci and their chromosome location are presented in Tables 2 and 3, respectively. A wide range of genetic diversity was detected in the panel of studied barley genotypes by gSSRs compared with EST-SSRs as revealed by a number of alleles (Na), number of effective alleles (Ne), Shannon's index (I), expected heterozygosity (He) and polymorphic information content (PIC). The 77 EST-SSRs detected 262 alleles in the studied genotypes, compared to 429 alleles for gSSRs. For EST-SSR, the number of alleles varied from 2 to 9 (SCSSR9398 on 6H) with a mean value of 3.40 ± 0.18 alleles/locus. The number of alleles amplified by gSSR primer pair was significantly higher and 429 alleles were amplified at 72 gSSR loci. The number of alleles varied from 2 to 15 (Bmac32 on 1H) with a mean value of 5.96 ± 0.34 alleles/locus. Out of 77 EST-SSR, 25 amplified a low number of alleles (2 alleles) compared with 11 of the gSSRs. Seventeen of the EST-SSRs amplified the relatively high number of alleles (5–9) compared with 43 of the gSSRs. The mean of effective number of alleles was 2.28 ± 0.12 with a range of 1.09 (GBM1404) to 7.00 (SCSRR9398) and 3.83 ± 0.23 with a range of 1.12 (EBmac659) to 10.65 (EBmac788) for EST-SSRs and gSSRs, respectively. Shannon's index ranged from 0.18 (GBM1404) to 2.05 (SCSRR9398) with an average of 0.87 ± 0.05 for the EST-SSRs compared to 0.21 (EBmac659) to 2.67 (Bmac40) with a mean value of 1.32 ± 0.06 for the gSSRs. A wide range of He and PIC values was observed in the set of gSSR compared with EST-SSR. The 72 SSRs had an average He and PIC of 0.63 ± 0.02 and 0.58 ± 0.02, respectively, indicating high efficiency in distinguishing genetic variation in the genotypes under study compared with the EST-SSRs with mean He and PIC of 0.48 ± 0.02 and 0.43 ± 0.02, respectively.

**Table 1:** Markers distribution and statistics concerning the selected genomic region.

Chromosome	Number of markers	Length (cM)	Interval spanned (cM)	Mean number of alleles	PIC
1H	20	125.89	6.29	4.90	0.48
2H	26	161.48	6.21	3.81	0.44
3H	19	137.70	8.10	4.84	0.57
4H	23	133.40	5.80	6.04	0.62
5H	23	166.40	7.56	3.69	0.46
6H	15	121.56	8.10	4.80	0.51
7H	20	152.60	7.63	4.90	0.48
Total	146	999.03	6.93	4.65	0.51

*PIC* polymorphic information content.

**Table 2:** Description of the used EST-SSR loci including chromosome location (Ch), major allele frequency (MAF), number of alleles (Na), effective number of alleles (Ne), Shannon’s information index (I), gene diversity (He) and polymorphic information (PIC).

Marker	Ch	MAF	Na	Ne	I	He	PIC	Marker	Ch	MAF	Na	Ne	I	He	PIC
GBM1143	1H	0.46	4	3.87	1.51	0.65	0.59	GBM1450	3H	0.47	3	2.35	0.93	0.57	0.48
GBM1216	1H	0.57	2	1.96	0.68	0.49	0.37	SCSSR1559	3H	0.51	5	2.95	1.30	0.66	0.62
GBM1336	1H	0.56	3	2.07	0.79	0.52	0.41	SCSSR25538	3H	0.81	2	1.44	0.49	0.31	0.26
GBM1411	1H	0.97	2	1.25	0.35	0.06	0.06	SCSSR25691	3H	0.52	4	2.72	1.15	0.63	0.58
GBM1451	1H	0.68	6	2.05	1.10	0.51	0.49	GBM1299	4H	0.89	3	1.25	0.42	0.20	0.19
GBM1461	1H	0.36	5	3.79	1.40	0.74	0.69	GBM1221	4H	0.36	7	3.90	1.54	0.74	0.70
GBM1480	1H	0.76	2	1.58	0.55	0.37	0.30	GBM1388	4H	0.69	2	1.74	0.62	0.43	0.34
SCSSR04163	1H	0.65	5	2.12	1.02	0.53	0.48	GBM1422	4H	0.57	4	2.51	1.10	0.60	0.55
GBM1149	2H	0.90	2	1.23	0.33	0.19	0.17	GBM1482	4H	0.44	6	3.81	1.45	0.72	0.68
GBM1208	2H	0.39	3	2.96	1.09	0.66	0.59	GBM1525	4H	0.71	3	1.84	0.80	0.46	0.41
GBM1251	2H	0.63	3	2.02	0.83	0.50	0.43	SCSSR14079	4H	0.39	5	3.77	1.45	0.73	0.69
GBM1309	2H	0.55	3	2.38	0.96	0.58	0.50	SCSSR18005	4H	0.49	3	2.15	0.82	0.53	0.43
GBM1365	2H	0.51	2	2.00	0.69	0.50	0.37	SCSSR20569	4H	0.46	5	2.84	1.18	0.65	0.58
GBM1366	2H	0.89	2	1.25	0.35	0.20	0.18	GBM5939	5H	0.54	3	2.19	0.86	0.54	0.45
GBM1408	2H	0.94	2	1.12	0.22	0.10	0.10	GBM1176	5H	0.83	3	1.41	0.56	0.29	0.27
GBM1459	2H	0.58	2	1.96	0.68	0.49	0.37	GBM1293	5H	0.67	3	1.98	0.86	0.49	0.44
GBM1468	2H	0.72	3	1.80	0.79	0.45	0.40	GBM1295	5H	0.72	2	1.68	0.59	0.40	0.32
SCSSR3381	2H	0.27	5	4.32	1.51	0.77	0.73	GBM1398	5H	0.80	2	1.48	0.51	0.33	0.27
SCSSR8447	2H	0.69	3	1.88	0.82	0.47	0.42	GBM1426	5H	0.64	2	1.86	0.65	0.46	0.35
SCSSR12344	2H	0.65	3	2.08	0.89	0.52	0.46	GBM1436	5H	0.79	3	1.53	0.65	0.35	0.32
GBM1110	3H	0.55	3	2.36	0.95	0.58	0.50	GBM1438	5H	0.49	3	2.52	0.99	0.60	0.52
GBM1139	3H	0.60	2	1.93	0.67	0.48	0.37	GBM1463	5H	0.80	3	1.50	0.61	0.33	0.30
GBM1159	3H	0.62	3	2.20	0.93	0.54	0.49	GBM1470	5H	0.49	3	2.20	0.86	0.55	0.44
GBM1405	3H	0.64	3	2.35	0.93	0.52	0.46	GBM1506	5H	0.38	5	3.40	1.35	0.71	0.65
GBM1413	3H	0.41	6	3.87	1.51	0.74	0.70	GBM5028	5H	0.60	2	1.92	0.67	0.48	0.36
SCSSR2306	5H	0.66	3	2.02	0.87	0.51	0.45	GBM1126	7H	0.51	3	2.11	0.80	0.53	0.42
SCSSR2503	5H	0.69	2	1.76	0.66	0.43	0.34	GBM1297	7H	0.94	2	1.13	0.24	0.12	0.11
SCSSR3907	5H	0.30	7	5.18	1.75	0.81	0.78	GBM1419	7H	0.94	3	1.14	0.26	0.12	0.12
SCSSR10148	5H	0.45	8	3.81	1.65	0.74	0.71	GBM1428	7H	0.56	2	1.97	0.69	0.49	0.37
SCSSR15334	5H	0.41	5	3.12	1.3	0.68	0.62	GBM1432	7H	0.87	2	1.29	0.38	0.22	0.20
SCSSR18076	5H	0.83	2	1.39	0.46	0.28	0.24	GBM1464	7H	0.51	6	3.04	1.37	0.67	0.63
GBM175	6H	0.65	3	2.02	0.85	0.50	0.44	GBM1472	7H	0.88	2	1.27	0.37	0.21	0.19
GBM1212	6H	0.87	3	1.31	0.48	0.24	0.23	GBM1516	7H	0.65	4	2.05	0.92	0.51	0.46
GBM1267	6H	0.84	2	1.38	0.44	0.27	0.24	SCSSR15864	7H	0.44	4	3.35	1.30	0.70	0.65
GBM1276	6H	0.61	3	2.17	0.90	0.54	0.47	SCSSR7970-1	7H	0.59	5	2.26	1.04	0.56	0.49
GBM1400	6H	0.88	3	1.28	0.45	0.22	0.21	SCSSR7970-2	7H	0.54	4	2.60	1.10	0.62	0.56
GBM1404	6H	0.96	2	1.09	0.18	0.08	0.08	GBM1552	–	0.79	2	1.49	0.51	0.33	0.27
SCSSR5599	6H	0.41	4	3.35	1.29	0.70	0.65	Mean		0.56	3.4	2.28	0.87	0.48	0.43
SCSSR9398	6H	0.21	9	7.00	2.05	0.86	0.84	SE	–	0.02	0.18	0.12	0.05	0.02	0.02
GBM1116	7H	0.86	2	1.33	0.41	0.25	0.22

**Table 3:** Description of the used genomic SSR loci including chromosome location (Ch), major allele frequency (MAF), number of alleles (Na), effective number of alleles (Ne), Shannon’s information index (I), gene diversity (He) and polymorphic information (PIC).

Marker	Ch	MAF	Na	Ne	I	He	PIC	Marker	Ch	MAF	Na	Ne	I	He	PIC
Bmac32	1H	0.15	15	10.06	2.43	0.90	0.89	HVM36	2H	0.62	3	2.00	0.80	0.50	0.41
Bmac63	1H	0.16	9	7.85	2.11	0.87	0.86	Bmac209	3H	0.36	7	3.80	1.53	0.74	0.70
Bmac213	1H	0.20	10	7.96	2.18	0.87	0.86	Bmag013	3H	0.35	6	3.57	1.41	0.72	0.67
Bmag149	1H	0.53	5	2.56	1.08	0.61	0.54	EBmac225	3H	0.18	11	8.24	2.23	0.88	0.87
Bmag211	1H	0.38	5	3.43	1.34	0.71	0.65	EBmac541	3H	0.51	6	2.97	1.31	0.66	0.62
Bmag45	1H	0.49	5	2.51	1.04	0.60	0.52	EBmac871	3H	0.23	8	6.23	1.94	0.84	0.82
Bmag82	1H	0.47	7	3.45	1.51	0.71	0.68	GBMS183	3H	0.48	5	3.22	1.37	0.69	0.65
Bmag504	1H	0.57	3	2.01	0.75	0.50	0.39	GMS116	3H	0.18	10	8.05	2.17	0.88	0.86
EBmac560	1H	0.74	2	1.64	0.58	0.39	0.31	HV13	3H	0.67	3	1.93	0.82	0.48	0.42
EBmac659	1H	0.94	2	1.12	0.21	0.10	0.10	HV13GEIII	3H	0.65	3	2.02	0.85	0.50	0.44
HVM20	1H	0.79	2	1.49	0.51	0.33	0.27	HVES1A	3H	0.73	2	1.65	0.59	0.40	0.32
HVM43	1H	0.94	4	1.12	0.27	0.11	0.11	Bmac310	4H	0.39	8	4.39	1.71	0.77	0.75
Bmac132	2H	0.80	2	1.47	0.50	0.32	0.27	Bmag375	4H	0.50	3	2.68	1.04	0.63	0.56
Bmac134	2H	0.59	8	2.62	1.38	0.62	0.59	Bmag419	4H	0.29	8	5.47	1.86	0.82	0.79
Bmag114	2H	0.82	2	1.43	0.48	0.30	0.26	Bmag740	4H	0.34	14	5.03	2.01	0.80	0.78
Bmag140	2H	0.52	3	2.52	1.08	0.60	0.53	Bmag138	4H	0.57	8	2.82	1.47	0.65	0.62
Bmag378	2H	0.52	3	2.59	1.02	0.61	0.54	EBmac635	4H	0.29	10	6.37	2.06	0.84	0.83
Bmag518	2H	0.48	5	3.04	1.30	0.67	0.62	EBmac788	4H	0.14	12	10.65	2.42	0.91	0.90
Bmag720	2H	0.18	11	7.37	2.13	0.86	0.85	EBmac906	4H	0.29	8	4.75	1.70	0.79	0.76
Bmag813	2H	0.31	9	5.38	1.87	0.81	0.79	EBmac679	4H	0.23	10	6.88	2.06	0.85	0.84
EBmac525	2H	0.71	3	1.76	0.71	0.43	0.36	GMS89	4H	0.35	5	3.55	1.37	0.72	0.67
EBmac558	2H	0.92	2	1.16	0.27	0.14	0.13	HVBAMY	4H	0.57	3	2.36	0.97	0.58	0.51
EBmac854	2H	0.74	4	1.71	0.80	0.41	0.38	HVM68	4H	0.47	5	2.79	1.17	0.64	0.58
EBmatc39	2H	0.71	2	1.70	0.60	0.41	0.33	HVM40	4H	0.31	5	3.99	1.46	0.75	0.71
GBMS0160	2H	0.39	9	4.67	1.85	0.79	0.77	HVM51	4H	0.61	2	1.91	0.67	0.48	0.36
Bmac96	5H	0.54	4	2.70	1.17	0.63	0.58	Bmac167	7H	0.55	3	2.28	0.92	0.56	0.48
Bmac113	5H	0.28	6	4.80	1.66	0.79	0.76	Bmag507	7H	0.15	14	9.74	2.41	0.90	0.89
Bmac163	5H	0.84	4	1.38	0.57	0.28	0.26	Bmag516	7H	0.29	8	5.85	1.90	0.83	0.81
Bmag751	5H	0.38	5	3.65	1.42	0.73	0.68	EBmag794	7H	0.80	3	1.51	0.63	0.34	0.31
Bmag812	5H	0.69	5	1.96	0.99	0.49	0.46	EBmatc16	7H	0.21	11	6.53	2.01	0.85	0.83
Bmac40	6H	0.24	12	7.93	2.67	0.87	0.86	GBMS141	7H	0.23	10	6.84	2.08	0.85	0.84
Bmag9	6H	0.30	5	3.90	1.44	0.74	0.70	GMS46	7H	0.70	3	1.87	0.82	0.46	0.42
Bmag807	6H	0.63	5	2.22	1.07	0.55	0.50	HVM49	7H	0.38	7	3.33	1.44	0.70	0.65
EBmac624	6H	0.48	3	2.69	1.04	0.63	0.55	HVLMNO1A	-	0.87	4	1.31	0.51	0.24	0.22
GBMS180	6H	0.35	6	4.47	1.63	0.78	0.74	MGB384	–	0.62	2	1.90	0.67	0.47	0.36
HVM31	6H	0.54	2	1.99	0.69	0.50	0.37	Mean		0.48	5.96	3.83	1.32	0.63	0.58
HVM74	6H	0.22	10	7.07	2.10	0.86	0.84	SE	–	0.02	0.34	0.23	0.06	0.02	0.02

### Genetic diversity across origins, growth habits and number of ear rows groups

To compare among groups' genetic variation based on gSSR and EST-SSR, the barley genotypes were grouped based on their (i) origins and breeding history, (ii) growth habits and (iii) the number of ear rows. Genetic diversity parameters for these groups are presented in Table 4. The mean number of different alleles (Na), effective number of alleles (Ne), Shannon's index (I), unbiased expected heterozygosity (uHe) and number of private alleles (Npa) calculated based on the allelic variation of 77 EST-SSRs were slightly higher for Iranian landraces (3.29, 2.18, 0.83, 0.47 and 5, respectively) compared with exotic landraces (3.20, 2.10, 0.80, 0.46 and 4, respectively), but the differences were not significant (Wilcoxon test, p-value = 0.10). However, the mean of all parameters was lower for "varieties and advanced breeding lines" (2.71, 1.86, 0.66, 0.39 and 2, respectively) compared with the landraces. Comparison of the parameters calculated based 72 gSSR data showed that the Iranian landraces and exotic landraces were equivalent in mean values of Na, Ne, I and uHe, but the number of private alleles in Iranian landraces (32 alleles) was significantly higher compared with exotic landraces (11 alleles) (Wilcoxon test, p-value ≤ 0.001). For this marker set, "varieties and advanced breeding lines showed the lower mean value of Na (4.12), Ne (2.73), I (1.05), and (0.56) except Npa which was 12 compared with 11 for exotic landraces.

**Table 4:** Allelic pattern of EST-SSR and gSSR markers across barley genotypes grouped based on origin, growth habit and number of ear rows.

	NS	Na		Ne		I		uHe		Npa	
Origin/type		EST-SSR	gSSR	EST-SSR	gSSR	EST-SSR	gSSR	EST-SSR	gSSR	EST-SSR	gSSR
Iranian landraces	69	3.29 (0.17)	5.53 (0.38)	2.18 (0.11)	4.07 (0.23)	0.83 (0.04)	1.25 (0.07)	0.47 (0.02)	0.61 (0.03)	5	32
Exotic landraces	50	3.20 (0.15)	5.17 (0.33)	2.10 (0.10)	3.88 (0.21)	0.80 (0.04)	1.20 (0.06)	0.46 (0.02)	0.60 (0.02)	4	11
Cultivars and advanced lines	25	2.71 (0.14)	4.12 (0.27)	1.86 (0.08)	2.73 (0.16)	0.66 (0.05)	1.05 (0.05)	0.39 (0.03)	0.56 (0.02)	2	12
Spring	61	3.19 (0.16)	5.25 (0.33)	1.92 (0.09)	3.10 (0.24)	0.72 (0.04)	1.12 (0.07)	0.41 (0.02)	0.55 (0.03)	3	17
Winter	69	3.31 (0.17)	5.64 (0.38)	2.25 (0.12)	3.85 (0.26)	0.85 (0.05)	1.32 (0.07)	0.49 (0.02)	0.65 (0.02)	14	44
Facultative	14	2.42 (0.12)	3.39 (0.17)	1.77 (0.09)	2.36 (0.15)	0.59 (0.05)	0.86 (0.06)	0.36 (0.03)	0.48 (0.03)	–	–
Two-row	79	3.21 (0.16)	5.37 (0.35)	1.94 (0.10)	3.06 (0.23)	0.72 (0.04)	1.10 (0.07)	0.41 (0.02)	0.54 (0.03)	8	13
Six-row	65	3.29 (0.17)	5.58 (0.37)	2.22 (0.11)	3.77 (0.24)	0.84 (0.04)	1.31 (0.07)	0.48 (0.02)	0.65 (0.02)	14	41

*NS* number of sample, *Na* number of different alleles, *Ne* effective number of alleles, *I* Shannon's information index, *uHe* Unbiased expected heterozygosity, *Npa* private alleles.

The mean values of Na, Ne, I and uHe estimated based on EST-SSR data were slightly higher in winter growth habit (3.31, 2.25, 0.85 and 0.49, respectively) than spring growth habit (3.19, 1.92, 0.72 and 0.41, respectively), but significantly higher than facultative genotypes (2.42, 1.77, 0.59 and 0.36, respectively) (Wilcoxon, p-values ≤ 0.01). A total of 14, 3 and zero EST-SSR private alleles were detected in winter, spring and facultative growth habits, respectively. Higher diversity was observed among the genotypes with various growth habits using gSSR markers compared with EST-SRRs. The mean values of Na, Ne, I, uHe and Npa in winter growth habit genotypes were 5.64, 3.85, 1.32, 0.65, and 44, respectively, compared with 5.25, 3.10, 1.12, 0.55 and 17, respectively, in spring growth habit and 3.39, 2.36, 0.86, 0.48 and zero, respectively, in facultative genotypes.

Considering ear row number, mean Na, Ne, I, uHe and Npa of EST-SSRs in two-row genotypes were 3.21, 1.94, 0.72, 0.411 and 8, compared with 3.29, 2.22, 0.84, 048 and 14, respectively in six-row genotypes. The mean of these parameters for gSSRs were 5.37, 3.06, 1.10, 0.54 and 13, in two-row barley and 5.58, 3.77, 1.31, 0.65 and 41, respectively in six-row barley. The analysis revealed slightly higher genetic diversity in six-row genotypes compared with two-row genotypes, but the difference was only significant for Npa. However, allelic diversity calculated based on gSSR data was higher in both groups compared with EST-SSRs.

### Inferring the population structure

**Figure 1:**
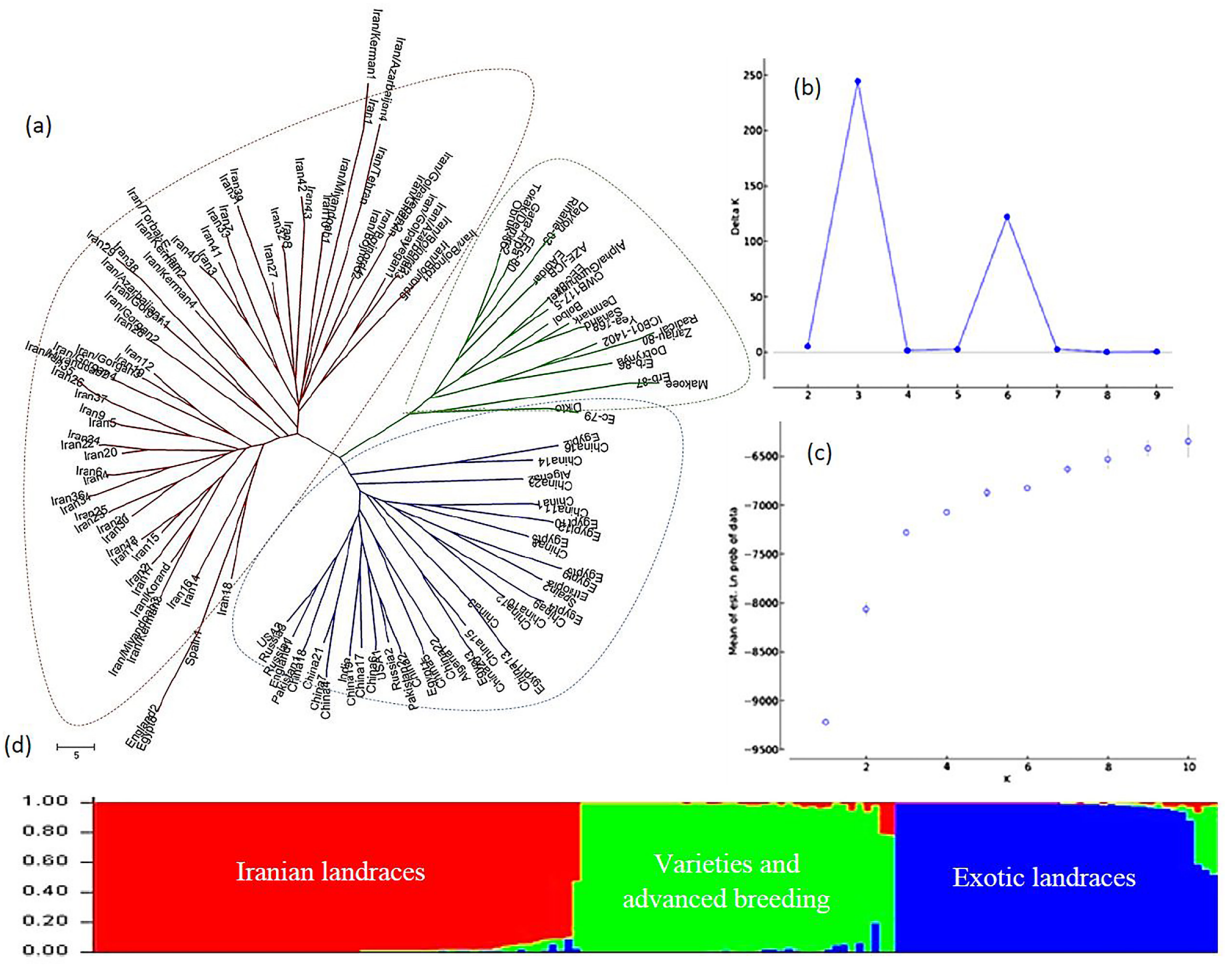
The genetic relationships and population structure of the 144 barley genotypes using EST-SSR data inferred by distance and model based cluster analyses. (**a**) Neighbor-Joining cluster analysis, (**b**) Delta K vs. K plotted for determining optimal numbers of subpopulations (K), (**c**) Mean Log probability values (LnP(D)) plotted as function of K (number of clusters) and (**d**) Estimated population structure on K = 3. Neighbor-Joining and model- based cluster analyses cold assign “Iranian landraces”, “exotic landraces” and “varieties and advanced breeding lines” into separate groups.

**Figure 2:**
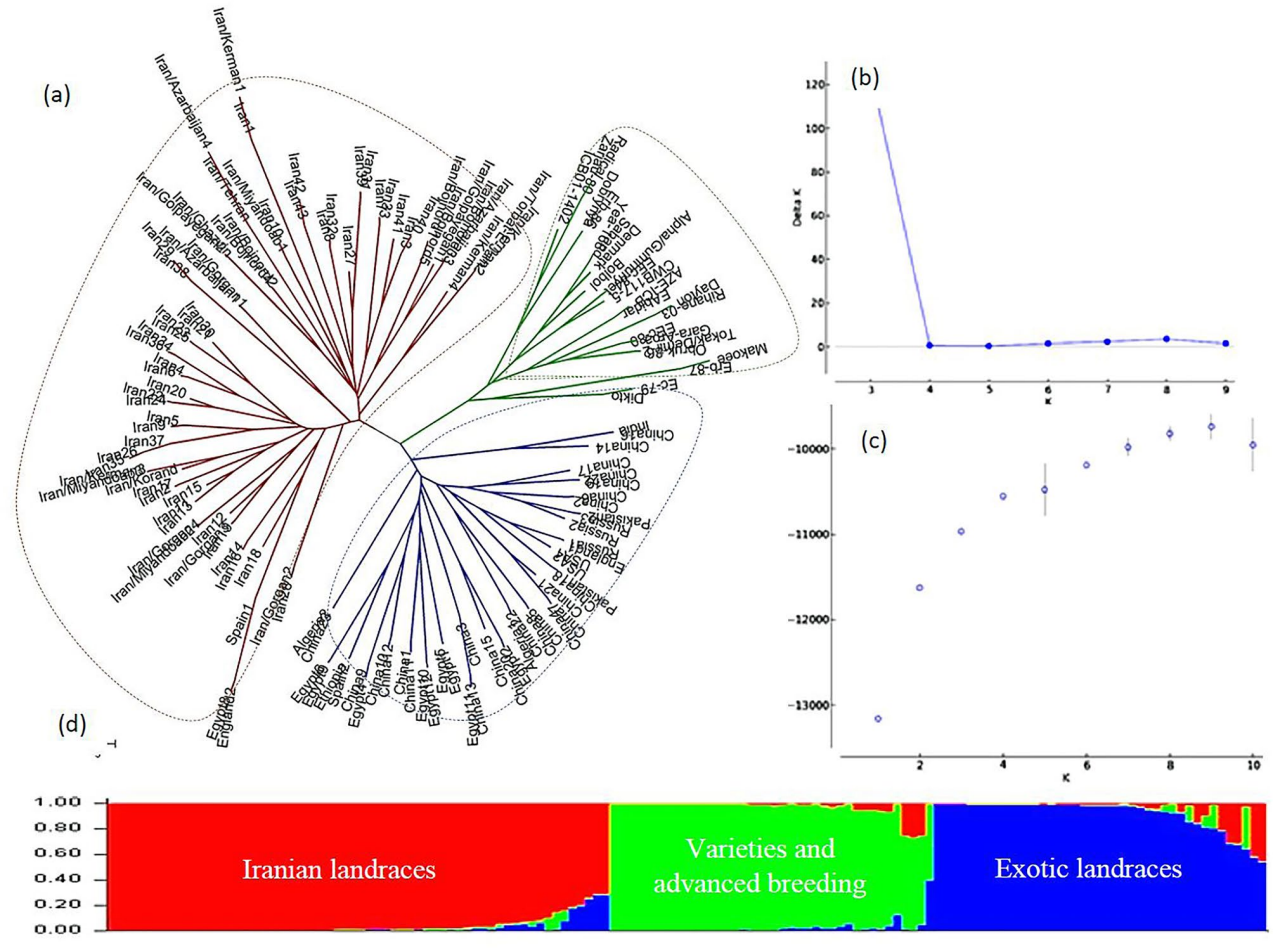
The genetic relationships and population structure of the 144 barley genotypes using SSR data inferred by distance and model based cluster analyses. (**a**) Neighbor-Joining cluster analysis, (**b**) Delta K vs. K plotted for determining optimal numbers of subpopulations (K), (**c**) Mean Log probability values (LnP(D)) plotted as function of K (number of clusters) and (**d**) Estimated population structure on K = 3. Neighbor-Joining and model- based cluster analyses cold assign “Iranian landraces”, “exotic landraces” and “varieties and advanced breeding lines” into separate groups.

**Figure 3:**
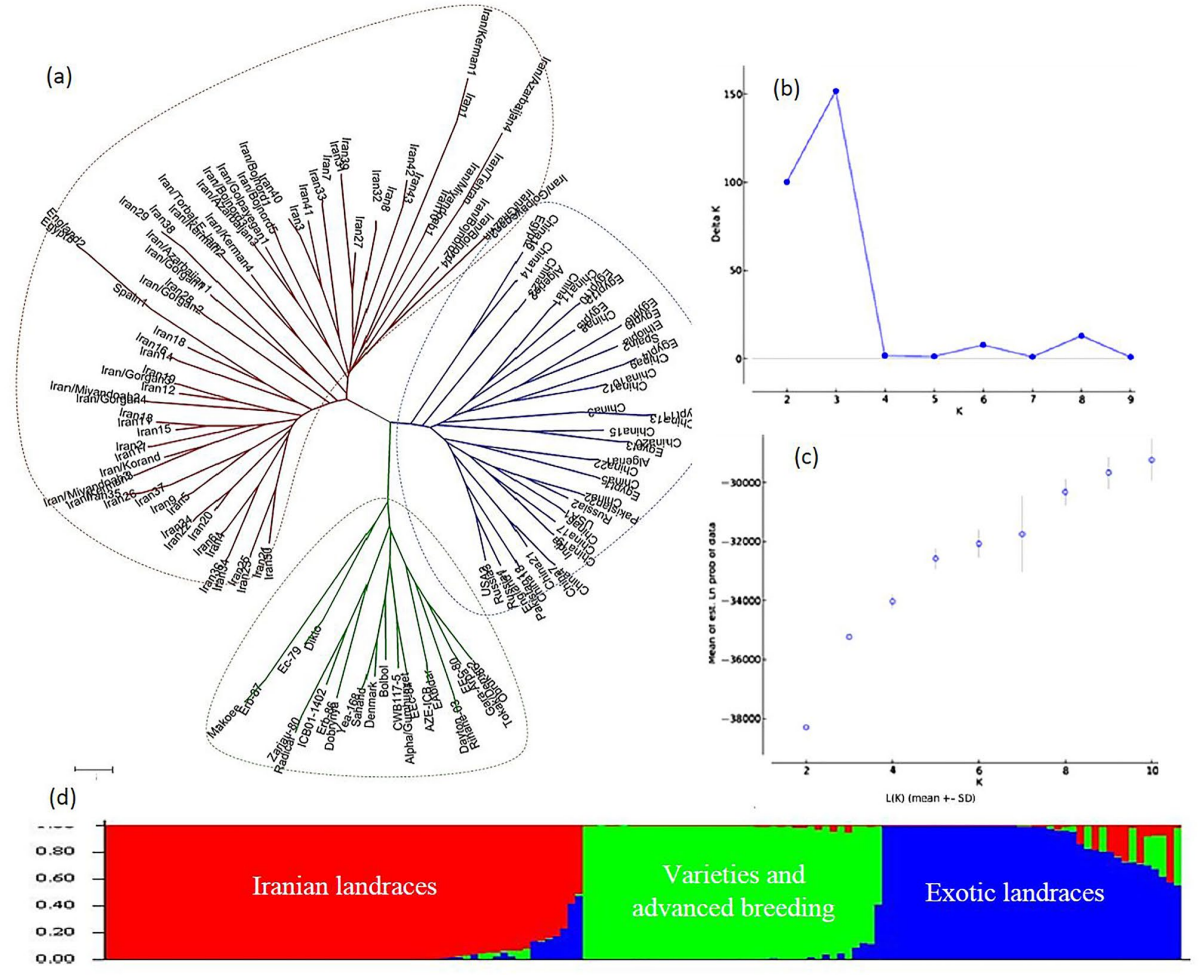
The genetic relationships and population structure of the 144 barley genotypes using EST-SSR and SSR data inferred by distance and model based cluster analyses. (**a**) Neighbor-Joining cluster analysis, (**b**) Delta K vs. K plotted for determining optimal numbers of subpopulations (K), (**c**) Mean Log probability values (LnP(D)) plotted as function of K (number of clusters) and (**d**) Estimated population structure on K = 3. Neighbor-Joining and model- based cluster analyses cold assign “Iranian landraces”, “exotic landraces” and “varieties and advanced breeding lines” into separate groups.

In the model-based clustering using SSR, EST-SSR, and combined data sets, the log-likelihood value [LnP(D)] increased continuously as K changed from 1 to 10, but inflection was evident when K increased from 2 to 3 (Figs. 1c, 2c, 3c). Thus, the most likely numerical value of K was 3. The further validation of the optimal number of clusters (K) was assessed using the second-order statistics of ΔK. The ΔK value showed a peak at K = 3 (Figs. 1b, 2b, 3b), which supported the classification of the studied populations into three major sub-populations corresponding to Iranian landraces, exotic landraces and "varieties and advance breeding lines" (Figs. 1d, 2d, 3d). Considering a probability of membership threshold of 70%, Iranian landraces, exotic landraces, and "varieties and advanced breeding lines" except few exceptions were assigned into three distinct sub-populations.

The distance-based cluster analysis using Neighbor-Joining algorithms and based on gSSR, EST-SSR, and combined data sets assigned the 144 barley genotypes into three clusters which were fully in agreement with the results of model-based clustering implemented in STRUCTURE (Figs. 1a, 2a, 3a). In the resulted phylogenetic trees, the Iranian landraces were grouped along with two landraces from Egypt and one from Spain. All the varieties and advanced breeding lines constructed a separate group and the exotic landraces were assigned into a distinct cluster.

**Figure 4:**
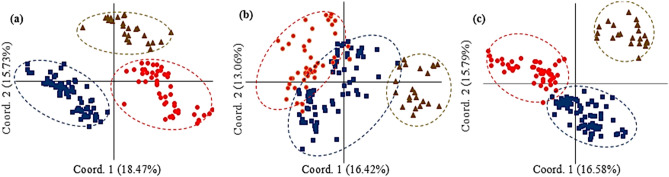
Principal coordinate analysis (PCoA) of 144 barley genotypes based on origins and breeding history [(blue diamonds) Iranian landraces, (red squares) exotic landraces and (brown triangles) cultivars and advanced breeding lines]. (**a**) EST-SSR, (**b**) gSSR and (**c**) EST-SSR + gSSR data.

**Figure 5:**
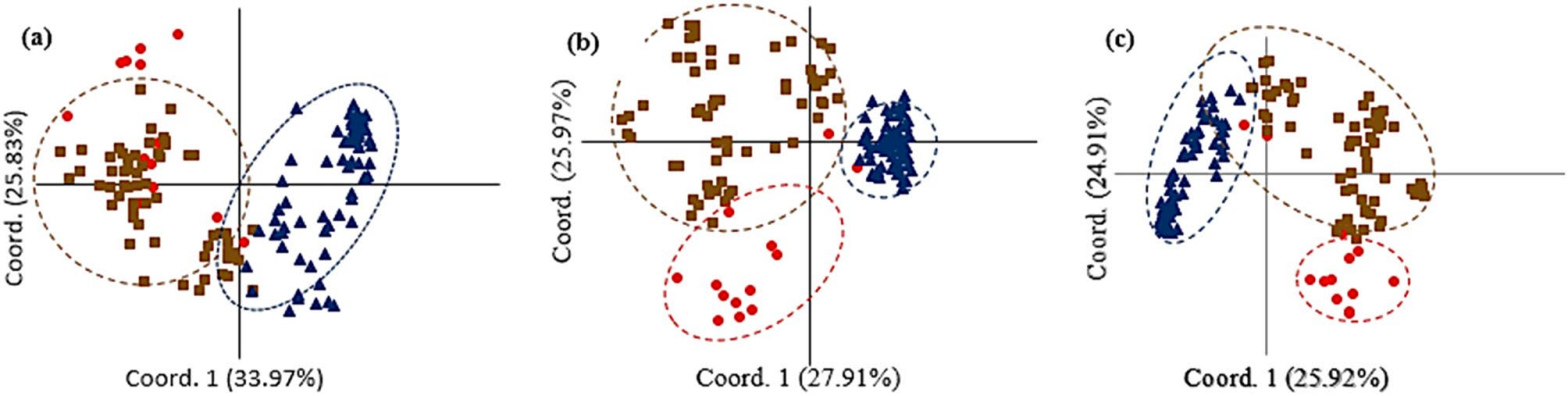
Principal coordinate analysis (PCoA) of 144 barley genotypes based on growth habits [(red diamonds) facultative, (brown squares) spring and (blue triangles) winter]. (**a**) EST-SSR, (**b**) SSR and (**c**) EST-SSR + SSR data.

**Figure 6:**
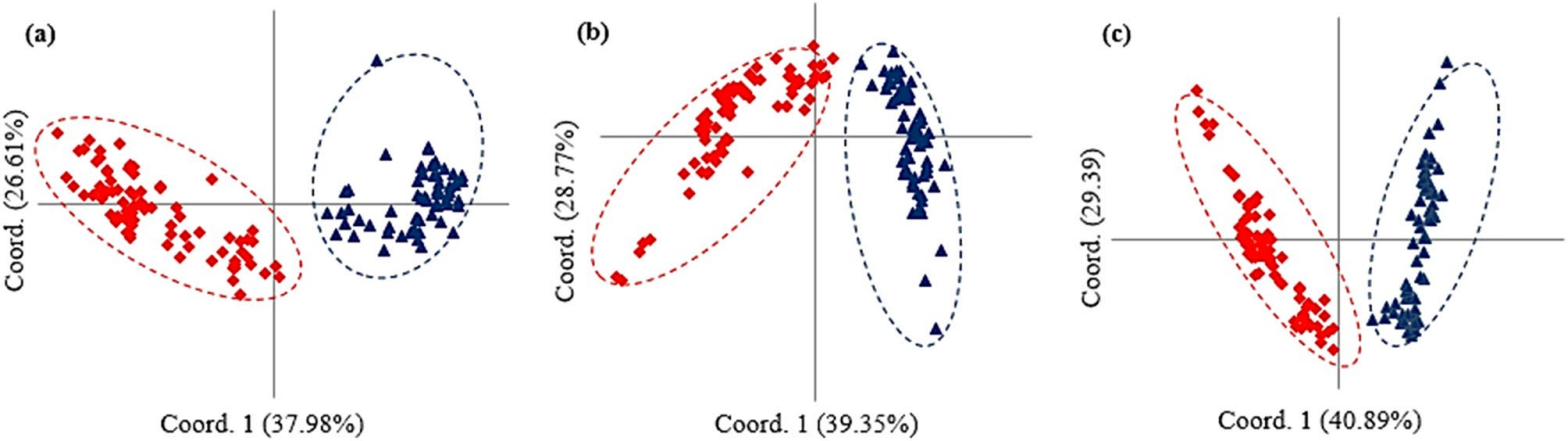
Principal coordinate analysis (PCoA) of 144 barley genotypes based on number of ear rows [(red diamonds) two- and (blue triangles) six-rowed]. (**a**) EST-SSR, (**b**) SSR and (**c**) EST-SSR + SSR data.

The PCoA based on origin and breeding history of the barley genotypes using EST-SSR data could separate Iranian landraces, exotic landraces, and "cultivars and advanced breeding lines" into distinct groups. The 1st and 2nd coordinates explained 18.47 and 15.73% molecular variation. The first coordinate discriminates Iranian landraces from the other genotypes (Fig. 4a). In PCoA using gSSR data, 16.42 and 13.06% of molecular variation conserved by the 1st and 2nd coordinates, respectively. Plotting of the genotypes based on two first coordinates revealed some admixture between Iranian and exotic landraces (Fig. 4b). When PCoA was performed using EST-SSR + gSSR data, the 1st and 2nd coordinates explained 16.58 and 15.79% of the variation, respectively and biplot of the genotypes using 1st and 2nd coordinates could discriminate all three groups (Fig. 4c). The principal coordinate analysis was also conducted to discriminate genotype based on their growth habits (spring, winter, and facultative). Using EST-SSR, the 1st coordinate explaining 37.97% of genetic variation distinguished winter habit versus the spring and facultative types, while based the 2nd coordinate accounting for 25.83% of the variation high admixture was observed among all growth habits (Fig. 5a). Analysis using gSSR and combination of EST-SSR, and gSSR data revealed better discrimination winter, spring and facultative genotypes. In both analyses, the 1st coordinate could separate wither types from spring and facultative genotypes and spring and facultative types were clustered into two distinct groups (Fig. 5b,c). Analysis of population structure between two- and six-row genotypes using PCoA based on EST-SSR, gSSR and EST-SSR + gSSR data showed discrimination of two groups in all analysis. The 1st coordinate accounting for 37.98, 39.35 and 40.89% of molecular variation of EST-SSR, gSSR and EST-SSR + gSSR data could distinguish two- and six-row barley (Fig. 6a–c).

The allelic pattern of EST-SSR and gSSR markers across barley genotype grouped based on origin and breeding history, growth habit, and number of ear rows are presented in Table 5. The number of different alleles per locus (Na) in Iranian landraces, exotic landraces and "cultivars and advanced breeding lines" were 3.29, 3.20 and 2.71 for EST-SSRs and 5.53, 5.17 and 4.12 for gSSRs, respectively. The effective number of alleles (Ne) was also higher for gSSR markers (4.07, 3.88 and 2.73) compared with EST-SSR marker (2.18, 2.10, and 1.86) across Iranian landraces, exotic landraces and "cultivars and advanced breeding liners". Shannon's index (I) and unbiased expected heterozygosity (uHe) ranged from 0.66 to 0.88 and 0.39 to 0.47, respectively for EST-SSR and 1.05 to 1.25 and 0.56 to 0.61 for gSSR markers. The number of private alleles was higher in Iranian landraces for both markers (5 and 32 for EST-SSR and gSSR, respectively) compared with exotic landraces (4 and 11 for EST-SSR and SSR, respectively) and "cultivars and advanced breeding lines (2 and 12 for EST-SSR and SSR, respectively).

**Table 5:** Distribution of 144 barley landraces, cultivars and advanced breeding lines used in this study according to their origin, number of ear rows and growth habit.

Origin/country	Two-rowed			Six-rowed			Sum
	W	S	F	W	S	F	
Iranian landraces	8	21	10	29	1	1	69
Exotic landraces							
China	2	8	2	9	2		23
Egypt		1		8	2	1	12
Algeria	1	1					2
Denmark		1					1
England		1		1			2
Ethiopia		1					1
India				1			1
Pakistan		2					2
Russia		3					3
Spain		1		1			2
USA		2					2
Cultivars and advanced breeding lines	2	13		7	2		24
Sum	13	55	12	56	7	2	144

*W* winter type, *S* spring type, *F* facultative type.

EST-SSR and gSSR diversity within spring barley as revealed by Na, Ne, I and uHe were not significantly differed from those of winter type but were higher compared with facultative genotypes. The higher number of private alleles were detected in winter genotypes (14 and 44 for EST-SSR and gSSR, respectively) compared with spring genotypes (3 and 17 for EST-SSR and gSSR, respectively) and facultative (no private allele). Genetic diversity based on ear row type revealed no major differences in the number of different alleles per locus and the effective number of alleles found in the two-row genotypes (EST-SSR: 3.21; gSSR: 5.37), (EST-SSR: 1.94; gSSR: 3.06) compared with the six-row genotypes (EST-SSR: 3.29, gSSR: 5.58), (EST-SSR: 2.22, gSSR: 3.77). But the genetic diversity as revealed by Shannon's index and unbiased expected heterozygosity was higher in the six-row genotypes (EST-SSR: 0.84; gSSR: 1.31) and (EST-SSR: 0.48; gSSR: 0.65) compared with the two-row genotypes (EST-SSR: 0.72; gSSR: 1.10) and (EST-SSR: 0.41; gSSR: 0.54). The six-row genotypes had also a higher number of private alleles (EST-SSR: 14; gSSR: 41) compared with the two-row Genotypes (EST-SSR: 8; gSSR: 13).

## Discussion

Knowledge of population structure and genetic relationships among the genotypes is a prerequisite for plant breeding programs as well as for emerging genome-wide association studies (GWAS) as an alternate approach for QTL detection in comparison to linkage map-based QTL analysis^23^. Besides, to achieve future production under changing environments, greater genetic diversity than that is present in current elite germplasms will be needed^24^. Fortunately, an extensive reservoir of biodiversity has been stored in genebanks as seeds of historical breeding materials, locally adapted landraces, and crop wild relatives which could be used to enrich cultivated gene pools. Although the ease of mobilization of favorable alleles into breeding materials is inversely related to the degree of adaptation, advances in genomics and molecular breeding technologies can accelerate the use of exotic germplasm for crop improvement^25^. However, access to novel allelic combinations available in the genebank collections requires thoughtful and renewed genotypic and phenotypic characterization of the materials^6^.

### Allelic variation and genetic diversity

In the present study, a panel of 144 barley genotypes (80 two-rowed and 64 six-rowed) including 69 landraces from various regions of Iran, and 50 landraces from other countries mainly from China and Egypt along with 9 advanced breeding lines and 16 commercial cultivars were genotyped using 149 SSR markers. This set of SSR markers amplified 691 alleles in the genotype under study and could provide a reasonable genotypic tool as a result of relatively high allele number, informativeness, and genomic coverage. The mean number of alleles/locus (4.64), gene diversity (0.55) and PIC (0.50) indicate a relatively high level of genetic variation in our study compared with some previous studies. An average of 3.20, 4.32, and 8.10 alleles/locus and the mean PIC of 0.38, 0.52, and 0.60 were reported in the analysis of diverse germplasms including wild and cultivated barley using SSR markers^26–28^.

The number of alleles/locus and PIC values have been suggested as a criterion to assess the level of genetic diversity in germplasm collections^29^. The presence of 17 SSR loci with PIC value of 0.80 or a higher and high number of alleles/locus could provide unique fingerprints for the studied barley genotypes and used for rapid analysis of genetic diversity and population structure in barley germplasm collections. A total of 691 polymorphic alleles detected in our study were enough to assess genetic relationships among barley landraces and cultivars. It was concluded that the presence of 350–550 alleles is enough for objective assessment of the genetic relationship between wheat accessions and it could be applicable for barley accessions as well^30,31^.

Overall genetic diversity in our study considering all types of materials was slightly lower compared with some studies carried out using barley landraces^18,28^. In our study, significant differences were observed between landraces and improved genotypes in number of alleles, number of effective alleles, number of private alleles, and gene diversity. On the other hand, the number of private alleles was 185 for the landraces while it was only 14 for cultivars and advanced breeding lines. It shows that modern breeding programs reduced the level of genetic variation in breeding materials. However, in our study, the number of landraces (119) was higher than that of cultivars and advanced breeding lines (25), but low level of genetic variation in the modern cultivars and breeding materials indicates the need of introducing new alleles from sources such as landraces and wild relatives to tackle climate change through plant breeding and better use of plant genetic resources. Compared with exotic landraces and "cultivars and advanced breeding lines", Iranian landraces had a higher level of gene diversity as revealed by Na, Ne, He, and number of private alleles. Significant differentiation assessed by F_ST_ (P = 0.001) was observed among the three groups. The number of private alleles in Iranian landraces (37 alleles at 29 loci) was also higher compared to exotic landraces (15 alleles at 14 loci) and “cultivars and advanced breeding lines” (14 alleles at 11 loci). Although Iranian landraces contained slightly more individuals compared with exotic landraces and “cultivars and advanced lines” (69 vs 50 and 25), this was consistent with the higher genetic variation in the Iranian landraces as revealed by number of alleles, number of effective alleles, gene diversity and Shannon’s index (Table 2). The higher genetic diversity in Iranian landraces could be due to the fact that a second domestication event may have occurred in barley, possibly in Central Asia at the eastern edge of the Iranian Plateau, and that this separate origin may have been the progenitor of present-day barleys found in East and South Asia^32,33^.

### Informative markers

Among the SSR loci with specific alleles in Iranian barley landraces, the following markers were associated with QTLs for malting quality, disease resistance, agronomic and physiological traits as reported by previous studies; Bmac209 (barley scald)^34^; Bmag345 (malting quality: hot water extract, diastatic power, alpha-amylase activity and free alpha-amino acid)^35^; Bmag382 (malting quality: hot water extract, grain protein content)^36^; Bmag518 (flag leaf physiological traits (intercellular CO_2_ concentration)^37^, chlorophyll content^38^, septoria speckled leaf blotch^39^, plump grain and 1000 grain weight^40^); Bmag740 (adult spot blotch and net type net blotch^39^ and days to maturity^41^); Bmag751 (carbon isotope discrimination and grain yield^41^); EBmac541 (dehydration gene for drought tolerance^42^); GBM1482 (lodging and lodging components^43^; GBMS183 (plant height and spike number per plant); GBMS141 (Endosperm hardness^40^); GMS89 (grain yield^26^); HVM40 (nitrate accumulation^44^, days to heading^26^) and HVM74 (major grain protein, soluble α-NH2 and grain protein concentration^44^. Assessment of group-specific alleles across the genome in plant germplasms should identify both the regions of the genome that should be conserved and the regions of the genome where there are opportunities to introgress new allelic diversity in the breeding materials without disrupting desirable gene complex.

### Population structure

We employed various statistical approaches to ensure the reliability of the inferences made regarding population structure in the collection. Although the results of model-based clustering were further ascertained by the results from the distance-based cluster analysis (Figs. 1, 2, 3) and PCoA (Figs. 4, 5, 6), the dendrograms resulted from NJ clustering were mostly in accord with the model-based clustering inferred groups. In the PCoA bi-plots, overlapping was observed among some of the inferred groups and represented a blurred distinction among the groups. Especially cluster I and III and cluster II and IV show some overlap. The row type in barley is an important determinant of the population structure^45^. In our studies, with K = 2, two- and six-rowed genotypes were assigned into distinct sub-populations. With an optimal number of clusters K = 4, except cluster III, two- and six-rowed genotypes were grouped separately. Cluster I, II, and IV consisted of 89%, 88% and 74% six-row, two-row, and two-row accessions, respectively. Cluster III is structured by 45% two-row and 55% six-row accessions. Our observations showed that Iranian barley landraces are different in comparison to the exotic landraces and mostly grouped separately. This could be due to the different evolutionary and domestication history of barley from Iran offers a plausible explanation for the observed differences^32,33^.

To further explore the genetic diversity and relationships among the clusters and within the clusters, allelic pattern and various diversity statistics were assessed for each of the STRUCTURE inferred clusters (Table 2). The cluster III (a relatively mixed group with two- and six-row accessions consisted of cultivars, advanced breeding lines and some landraces from various countries) with a higher number of member among clusters (42) had maximum mean number of alleles, number of effective alleles, gene diversity and Shannon index (4.29, 3.09, 0.57 and 1.09). Group-specific rare alleles detected in cluster III was also high compared with the others, emphasizing the presence of a higher diversity in this group. A total of 52 group-specific alleles at 36 loci were identified in cluster III. The number of group-specific alleles for cluster I was 19 alleles at 18 gSSR loci, one for group IV and no specific alleles were detected for cluster II. The higher heterogeneity in cluster III may be due to eco-geographical diversity and multiple barley domestication sites apart from Fertile Crescent. Comparison of the genetic structure of Western and Eastern cultivated barleys proposed a secondary domestication site of barley somewhere 1500–3000 km east of Fertile Crescent and found greater allelic differences between these groups^32^.

## Conclusion

In the present study, we utilized microsatellite markers to assess the effect of breeding history, origin, and growth type on genetic diversity and population structure of barley genotypes. Assigning of Iranian landraces, exotic landraces and "varieties and advanced breeding lines" into separate groups based on both gSSR and EST-SSt data could be due to their different evolutionary, domestication, and breeding history. The higher genetic diversity in Iranian landraces revealed by high allelic diversity and private alleles number could be due to the fact that a second domestication event may have occurred in barley, possibly in Central Asia at the eastern edge of the Iranian Plateau. The allelic diversity presented in the studied collection, especially in Iranian landraces was found to be correlated with population structure, domestication, and eco-geographical factors. The high allelic richness in the studied set of barley genotype as revealed by genetic and statistical analyses provides insights into the available diversity and allows construction of core groups based on maximizing allelic diversity for use in Iranian barley breeding programs. The Iranian landrace panel comprised lines that were well distributed across all eco-geographical regions with different climatic regimes which could be used for introgression of favorite alleles into breeding lines.

## Materials and methods

### Plant materials

A set of 144 barley genotypes (80 two-rowed and 64 six-rowed) including 69 landraces from various regions of Iran, 23 from China, 12 from Egypt and 15 from the USA, England, India, Pakistan and Algeria along with 9 advanced breeding lines and 16 commercial varieties was used. Out of 144 genotypes, 61, 69, and 14 were spring, winter, and facultative growth habits, respectively. The majority of six-row and two-row barley genotype were winter types (88.8%, 70.0%, respectively) (Table 5).

### Genomic and EST-SSR genotyping

Genomic DNA was extracted from bulked fresh leaf samples of each genotype using the CTAB method^46^. Although the genotypes used in our study are homozygous lines, a pool of leaves from 15 plants of each genotype was used for DNA extraction to provide a reliable measure of the possible genetic heterogeneity within each genotype^47,48^. The 0.8% (w/v) agarose gel electrophoresis and spectrophotometer were used to examine the quality and quantity of the DNA samples, respectively. Each genotype was analyzed using a set 77 and 72 polymorphic EST-SSR and gSSR markers, respectively distributed on seven chromosomes of barley. Bin locations of the markers were adapted from Grain Genes Map Data Report: Barley, Steptoe × Morex, SSR (https://wheat.pw.usda.gov/cgi-bin/graingenes/report). The PCR reaction mixture was prepared in a final volume of 10 μL containing 40 ng template DNA, 1 × PCR buffer, 0.025 units *Taq* DNA polymerase (Sinagene, Iran), 0.2 mmol dNTPs, and 0.8 pmol forward and reverse primers. Amplifications were performed under the following conditions: 4 min at 94 °C, followed by 35 cycles at 94 °C for 1 min, primer annealing at 58–68 °C for 1 min depending on primers and 72 °C for 1 min, and a final extension at 72 °C for 7 min. A Gel-Scan 3000 electrophoresis system (a real-time laser scanning electrophoresis system, Corbett Co.) based on 4% ultra-thin (0.2 mm) non-denature polyacrylamide gel stained by ethidium bromide was used to visualize PCR products.

### Statistical analysis

Number of allele (Na), number of effective allele (Ne), major allele frequency (MAF), Shannon's index (I), gene diversity (He) and polymorphic information content (PIC) were determined for all the analyzed markers across the total population as well as landraces and "varieties and advanced breeding lines" using PowerMarker version 3.25^49^ and GenAlEx 6.5 software^50^. Polymorphism Information Content (PIC) values were determined as $PIC = 1 - \sum {p_{i}^{2} } - 2\sum {p_{i}^{2} p_{j}^{2} }$^51^. Gene diversity was calculated as $He = 1 - \sum {p_{i}^{2} }$^52^. Shannon's information index was estimated as $I = - \sum {p_{i} \;lnp_{i} }$, where *p*_*i*_ and *p*_*j*_ are the frequencies of the *i*th and *j*th alleles of a given locus, respectively.

Population structure in a collection of 144 barley genotypes including Iranian and exotic landraces, advanced breeding lines, and cultivars was assessed by two statistical methods. The model-based clustering implemented in STRUCTURE 2.3.4^53^ was performed by running 100,000 Markov chain Monte Carlo (MCMC) iterations after a burn-in of 100,000 replicates with 5 independent runs per K ranging from 1 to 10, using the admixture model with correlated allele frequencies, which estimates fractions of individual genomes that belong to different ancestry groups. The optimal number of cluster (K) was determined by calculating the mean posterior probability for each K value, LnP(D), which is based on the estimated maximum log-likelihood values^53^. The ΔK values (the rate of change in the log probability of data between successive K values) was also calculated as suggested by^54^ using STRUCTURE-HARVESTER, version 0.6.94^55^.

We used principal coordinate analysis (PCoA) for genetic differentiation between two- and six-row genotypes, among spring, winter, and facultative genotypes as well as Iranian landraces, exotics landraces, and “varieties and advanced breeding lines. The PCoA was performed on GenAIEx 6.501 software^27^. Finally, the unrooted neighbor-joining (N-J) clustering algorithm under the Reynold 1983 distance coefficient was applied using the software PowerMarker 3.25^26^ to investigate the relationship of barley accessions.

A hierarchical analysis of molecular variance (AMOVA) implemented in GenAlex 6.501^27^ was used the partition of the observed molecular variation among and within the three clusters inferred using STRUCTURE and NJ cluster analyses corresponding to Iranian landraces, exotic landraces and "cultivars and advance breeding lines". The genetic parameters including the number of a different allele with a frequency ≥ 0.05, the number of effective alleles, Shannon's index (I), and unbiased expected heterozygosity (uHe = (2N/(2N − 1)) × He) were estimated for each cluster, where N is group size.

## Data Availability

The data are available on request.
